# Multi-Scale Associations between Vegetation Cover and Woodland Bird Communities across a Large Agricultural Region

**DOI:** 10.1371/journal.pone.0097029

**Published:** 2014-05-15

**Authors:** Karen Ikin, Philip S. Barton, Ingrid A. Stirnemann, John R. Stein, Damian Michael, Mason Crane, Sachiko Okada, David B. Lindenmayer

**Affiliations:** 1 Fenner School of Environment and Society, Australian Research Council Centre for Environmental Decisions, National Environmental Research Program Environmental Decisions Hub, The Australian National University, Canberra, Australia; Institute of Agronomy, University of Lisbon, Portugal

## Abstract

Improving biodiversity conservation in fragmented agricultural landscapes has become an important global issue. Vegetation at the patch and landscape-scale is important for species occupancy and diversity, yet few previous studies have explored multi-scale associations between vegetation and community assemblages. Here, we investigated how patch and landscape-scale vegetation cover structure woodland bird communities. We asked: (1) How is the bird community associated with the vegetation structure of woodland patches and the amount of vegetation cover in the surrounding landscape? (2) Do species of conservation concern respond to woodland vegetation structure and surrounding vegetation cover differently to other species in the community? And (3) Can the relationships between the bird community and the woodland vegetation structure and surrounding vegetation cover be explained by the ecological traits of the species comprising the bird community? We studied 103 woodland patches (0.5 - 53.8 ha) over two time periods across a large (6,800 km^2^) agricultural region in southeastern Australia. We found that both patch vegetation and surrounding woody vegetation cover were important for structuring the bird community, and that these relationships were consistent over time. In particular, the occurrence of mistletoe within the patches and high values of woody vegetation cover within 1,000 ha and 10,000 ha were important, especially for bird species of conservation concern. We found that the majority of these species displayed similar, positive responses to patch and landscape vegetation attributes. We also found that these relationships were related to the foraging and nesting traits of the bird community. Our findings suggest that management strategies to increase both remnant vegetation quality and the cover of surrounding woody vegetation in fragmented agricultural landscapes may lead to improved conservation of bird communities.

## Introduction

Agricultural landscapes worldwide share a common history of native vegetation modification due to intensive land use, including tropical forests of Brazil [1], sagebrush-steppe landscapes of northwest America [2], semi-natural grasslands of northern Europe [3], and temperate eucalypt-dominated woodlands of Australia [4]. Within such landscapes, the intensification and expansion of agriculture has led to widespread loss and fragmentation of native vegetation [5]. Native vegetation patches provide key habitat resources for many species, including those of conservation concern, helping these species to persist in fragmented agricultural landscapes. For example, previous studies have found that remnant native vegetation was crucial for mammals in southern Spain [6] and the western Great Plains of North America [7], declining birds in the United Kingdom [8], The Netherlands [9] and Australia [10], [11], and ant communities in Brazil [12]. It is therefore important to better understand the factors affecting biodiversity in native vegetation patches to inform conservation strategies in agricultural landscapes.

It is well documented that both patch and landscape vegetation cover and structure are important for woodland birds in agricultural landscapes [13]–[16]. Such previous research has focused predominantly on *species-specific* responses (e.g. individual species occupancy) or effects on *species diversity* (richness and abundance). How patch and landscape-scale vegetation affects bird *community composition* is comparatively less well-understood [17], [18]. Recent studies suggest that bird communities are influenced by vegetation at both the patch and landscape scale [10], [18]–[20], consistent with species-specific and species diversity investigations. There have been mixed findings, however, regarding the differing effects of vegetation at these scales on community composition [10], [18] and the stability of responses over time and space [17]. More community-level studies from different agricultural regions worldwide are needed to identify if these seemingly idiosyncratic findings can be integrated into global generalities [21]. Further, from a conservation perspective, community-level studies need to build on those of single species and species diversity within the same agricultural region, to integrate policy and management recommendations.

**Figure 1:**
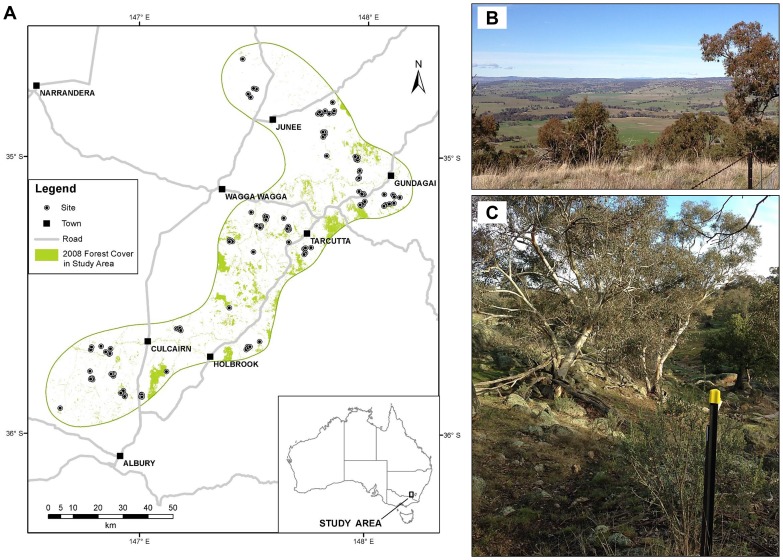
The South-West Slopes Restoration Study, New South Wales, Australia: (A) location of woodland sites across the region [note that site points are not drawn to scale], (B) native vegetation cover in the surrounding landscape, and (C) a survey site in a woodland patch. Images: K. Ikin.

We investigated how woodland bird communities were associated with patch and landscape scale vegetation across a large agricultural region. Our South-West Slopes Restoration Study is a spatially-extensive investigation in the ‘wheat-sheep belt’ of eastern Australia [22]. Across a region of approximately 6,800 km^2^, we have established 103 sites in woodland patches with varying amounts of woody vegetation cover in the surrounding landscape (Fig. 1). These sites were surveyed for birds and vegetation in 2002 and again in 2008. Montague-Drake et al. [11] found that the probability of detecting 13 bird species of conservation concern within these sites was related to a combination of patch-scale and landscape-scale vegetation cover and structure. Cunningham et al. [23] went on to establish that total bird species richness and the richness of bird species of conservation concern was related to native vegetation cover at multiple spatial scales in the surrounding landscape and over time. In a related study, Cunningham et al. [24] showed that individual species differed in their relationship with vegetation cover between spatial scales, and also to temporal changes in vegetation cover. These studies, however, did not consider the effect of multi-scale vegetation cover on the bird community from the perspective of community composition. The goal of our paper was to address this knowledge gap, and to this end, we investigated the following three key questions.

1. How is the bird community associated with the vegetation structure of woodland patches and the amount of vegetation cover in the surrounding landscape? We investigated patch-scale vegetation attributes known to be important for woodland bird occurrence [11]. These included habitat structures present in the ground-layer (grasses, leaf litter, moss and lichen), mid-layer (mid-sized trees, mid-storey cover) and canopy-layer (hollow-bearing trees, canopy depth) strata, as well as attributes capturing vegetation condition (tree dieback, mistletoe). We also investigated surrounding woody vegetation cover in the landscape at three scales of measurement known to be important for woodland bird richness and occurrence: 100 ha, 1,000 ha, and 10,000 ha [23], [24]. Our aim was to identify the relationships between multi-scale vegetation cover and bird community structure, and also to see if this relationship was consistent over time. To do so, we compared data from 2002 and 2008.
2. Do species of conservation concern respond to woodland vegetation structure and surrounding vegetation cover differently to other species in the community? Southeastern Australia supports a suite of woodland-dependent bird species that several authors have identified as being of conservation concern, due to declining abundance and occurrence and/or listing in national and state-level threatened species legislation, for example the *Environment Protection and Biodiversity Conservation Act 1999*. These species have been adversely affected by ongoing vegetation loss, fragmentation and degradation (reviewed by [25], but see [26], [27]). Some authors have suggested that species of conservation concern share similar life history attributes [28], [29], and can be distinguished from other species in the assemblage. For example, many woodland bird species considered to be in decline are ground-foraging insectivores [25], [30]. Other authors, however, have not identified clear relationships between long-term trends and life history attributes [26], [31]. For instance, Mac Nally et al. [32] found similar effects of long-term drought on all foraging, nesting and movement guilds. In our study, we investigated how species of conservation concern were associated with vegetation cover at multiple scales of measurement, and whether they could be grouped by their responses.
3. Can the relationships between the bird community and the woodland vegetation structure and surrounding vegetation cover be explained by the ecological traits of the species comprising the bird community? The occurrence of individual species at a given location is underpinned, in part, by their life history traits and how these traits dictate their resource requirements [33]–[36]. The community composition of a woodland patch, therefore, should reflect the relationship between the ecological traits (i.e. foraging methods and nesting strategies) of the species set and the woodland vegetation and surrounding vegetation cover. Thus, to deepen our understanding of the mechanisms by which the bird community is associated with woodland vegetation and surrounding vegetation [37], [38], we investigated the underlying relationships between individual species traits and patch and landscape vegetation cover. Further, by comparing these relationships between 2002 and 2008, we were able to determine if these relationships were consistent through time.

Answering these three questions will provide better understanding about what patch and landscape vegetation attributes in agricultural landscapes are most important for birds at the community-level, including species of conservation concern. This will lead to improved understanding of how to target management strategies in fragmented agricultural landscapes to improve conservation of woodland bird communities.

## Methods

### Ethics Statement

This field study was undertaken with animal ethics approval obtained through The Australian National University Animal Experimentation Ethics Committee. The study was conducted on privately-owned land and access permission was granted by landowners prior to establishing the field sites.

### Study Area

We conducted our study in the South-West Slopes bioregion of New South Wales, in southeastern Australia [22]. Native vegetation within the region is predominantly temperate eucalypt woodland, with approximately 15% of original vegetation cover remaining [39]. For this study, we focused on 103 woodland patches located on 35 farms within the region (Fig. 1). The patches included old growth (n = 59) and regrowth (n = 44) woodland, and ranged in area from 0.5 ha to 53.8 ha (mean 9.3 ha). All were located in mixed cropping/grazing landscapes. We established a permanent field site in the centre of each patch in 2002; these were separated by a minimum distance of 120 m (average 800 m). A site consisted of a 200 m transect with three survey points, located at the 0 m, 100 m and 200 m distances. We surveyed the birds and vegetation at each site in 2002 and 2008 (see below). During this time, southeastern Australia experienced the most severe drought recorded since 1900. The “Millennium Drought” spanned the period 2001–2009 [40], representing an unprecedented number of sequential years with below-median rainfall. The effects of this drought included a 45% reduction in stream flow [40] and increased tree mortality in dryland ecosystems [41].

### Woodland Patch Vegetation

We identified 11 attributes that are each important determinants of site occupancy for at least three woodland birds of conservation concern (Table 1; [11]), and measured these variables in each woodland site in 2002 and again in 2008. We established three 20×20 m vegetation plots per transect, centred at the 0, 100 and 200 m transect survey points. Within these, we counted the number of trees with mid-sized stems (15–30 cm diameter at breast height) and the number of trees with hollows (cavities) visible from the ground. We adjusted these values to the number of trees per hectare. We measured the depth of the canopy (length of canopy from the base of the crown to the tree tip) of the tallest tree and scored the level of tree dieback (ranging from no dieback to tree death). At the corners of each plot, we established four 1×1 m sub-plots (12 sub-plots per transect). Within these, we visually estimated the percent cover of leaf litter, annual grasses and native grasses. We then calculated site averages for each of these plot and sub-plot variables. Lastly, we recorded the presence of mistletoe (*Amyema miquelii* and *A. pendula*) and midstorey cover in the plots and the presence of moss and lichen in the subplots. We used paired t-tests to test for significant differences in each of the vegetation variables between 2002 and 2008 (all analyses were conducted using ‘R’, version 3.0.2, http://www.r-project.org/, unless otherwise specified). To account for the multiple tests, we used the Bonferroni correction, and considered differences to be significant when P≤0.003.

**Table 1:** Summary of woodland patch vegetation and percent woody vegetation cover variables for 2002 and 2008.

Variable	Description	2002 Range(mean)	2008 Range(mean)	P-Value
Mid-size trees	Number of trees with DBH 15–30 cm, adjusted to per ha	0.00–12.67 (2.69)	0.00–23.67 (2.63)	0.604
Canopy depth	Depth of the canopy of the tallest tree	3.00–20.00 (10.92)	5.67–24.00 (13.8)	<0.001
Hollow-bearing trees	Number of trees with visible hollows in/overhanging plots, adjusted to per ha	0.00–108.3 (25.93)	0.00–91.67 (16.99)	<0.001
Dieback score	Amount of tree dieback. Scores are 0 = no dieback,1 = branch tips dead, 2 = extensive defoliation,3 = epicormic growth, 4 = tree death	0.00–3.33 (1.20)	0.00–4.00 (1.74)	<0.001
Mistletoe	Presence of mistletoe	Present: 23 sites,Absent: 80 sites	Present: 18 sites,Absent: 85 sites	0.132
Midstorey cover	Presence of midstorey cover	Present: 54 sites,Absent: 49 sites	Present: 9 sites,Absent: 94 sites	<0.001
Strata	Number of strata	2.00–4.00 (2.86)	1.33–4.00 (2.39)	<0.001
Annual grasses	Percent annual grasses cover	0.00–85.00 (25.98)	0.00–80.83 (27.41)	0.307
Leaf litter	Percent leaf litter cover	0.42–81.67 (30.35)	0.42–77.5 (31.49)	0.474
Native grasses	Percent native grasses cover	0.00–34.17 (8.46)	0.00–62.08 (11.16)	0.056
Moss and lichen	Presence of moss and/or lichen cover	Present: 61 sites,Absent: 42 sites	Present: 59 sites,Absent: 44 sites	0.747
Woody vegetation cover,100 ha scale	Percent woody vegetation cover within 100 ha	0.00–43.86 (3.61)	0.00–44.21 (4.66)	<0.001
Woody vegetation cover,1,000 ha scale	Percent woody vegetation cover within 1,000 ha	0.00–37.48 (3.75)	0.03–37.91 (4.37)	<0.001
Woody vegetation cover,10,000 ha scale	Percent woody vegetation cover within 10,000 ha	0.05–23.77 (5.38)	0.09–24.79 (6.19)	<0.001

Paired t-tests were used to test for significant differences (P≤0.003) between the two study years.

We used Spearman’s Rank Correlation Coefficients to check for strong positive (r_s_ ≥0.5) or negative (r_s_ ≤ −0.5) correlations between the vegetation variables in three datasets: (1) 2002 and 2008 combined, (2) 2002 only, and (3) 2008 only. Where variables were correlated, we retained only one of them for the subsequent analyses. In the 2002 and 2008 combined dataset, we retained canopy depth, number of mid-sized trees and hollow-bearing trees, dieback score, leaf litter and native grass cover, and presence of mistletoe, midstorey cover, and moss and lichen. In the 2002 dataset, we retained canopy depth, number of mid-sized trees, hollow-bearing trees and strata, dieback score, leaf litter and native grass cover, and presence of mistletoe, and moss and lichen. In the 2008 dataset, we retained canopy depth, number of mid-sized trees, hollow-bearing trees and strata, dieback score, leaf litter and native grass cover, and presence of mistletoe and midstorey cover.

### Surrounding Woody Vegetation

We measured woody vegetation cover surrounding our 103 woodland sites in 2002 and 2008 at three scales of measurement of increasing orders of magnitude: 100 ha, 1,000 ha, and 10,000 ha (Table 1). Previous research within the study region has shown that bird species richness is related to vegetation cover at each of these scales [23]. To measure vegetation cover in each year, we used grids of Forest Extent (FE) derived from Landsat TM and MSS satellite imagery from 2002 and 2008 (see [42] for a detailed description of the satellite imagery specifications). Grid cells (25 m×25 m resolution) with a minimum canopy cover of 20% over a minimum area of 0.2 ha with a potential height of ≥2 m were characterised as woody vegetation; this classification included old-growth, regrowth and replanted vegetation. We then determined the number of grid cells with woody vegetation at each scale to calculate the percent woody vegetation cover.

In each of the three datasets (2002 and 2008 combined, 2002 only, and 2008 only), percent woody vegetation cover in the 100 ha and 1,000 ha scales were positively correlated, as were the 1,000 ha and 10,000 ha scales. To reduce this collinearity, we calculated new 100 ha and 1,000 ha woody vegetation cover variables. We did this by subtracting from the value of the smaller scale the value of the larger scale it was nested within, following the method of Rhodes et al. [43]:

- New 100 ha percent woody vegetation cover = original 100 ha percent cover – original 1,000 ha percent cover
- New 1,000 ha percent woody vegetation cover = original 1,000 ha percent cover – original 10,000 ha percent cover.

Thus, the recalculated percent woody vegetation cover variables equalled the difference between the original variable and the value of the larger scale that it is nested within, whilst the value of the 10,000 ha percent woody vegetation cover remained the same. We used Spearman’s Rank Correlation Coefficients to confirm that our new variables were not correlated. We used paired t-tests to test for significant differences (P≤0.003, Bonferroni correction) in each of the percent woody vegetation cover variables between 2002 and 2008.

### Birds

We surveyed each site for birds in the austral spring of 2002 and again in spring 2008. These surveys were completed in early November, which is the peak breeding season in the study region when most birds, including summer migrants, establish breeding territories and therefore exhibit high site fidelity [11]. In each year, we surveyed each survey point twice, totalling six surveys per site (three survey points per transect by two repeats). For each survey, observers stood at the survey point for five minutes and recorded as present all birds observed within 50 m of the point (excluding birds flying overhead). At the completion of the survey, the observer would move to the next survey point; once all points in the site were surveyed, the observer would move to a different site. Repeat surveys were undertaken by a different observer on a different day. This survey protocol of multiple observers and repeat visits overcomes observer heterogeneity effects [44] and helps to correct for false-negative errors, i.e. failure to detect a species that is present at the site [45]. All surveys were completed within four hours of first light.

We assessed the thoroughness of our bird surveys to ensure appropriate interpretation of our results [46]. To do this, we used multiple richness estimators to calculate separate total bird species richness estimates for 2002 and 2008 (EstimateS 9 [47]). We then compared the observed numbers of species with the estimated number of species. To avoid possible bias introduced by cryptic or wide-ranging species that were not detected in our surveys, we excluded waterbirds and species recorded at only one site from subsequent analyses.

#### Bird community composition (Questions 1 and 2)

We used a two-step process to analyse how the bird community was associated with woodland patch vegetation and surrounding woody vegetation in 2002 and 2008 combined, 2002 only and 2008 only. First, we determined whether there was structure in the bird community, i.e. whether the bird species could be characterised by the woodland patches where they occurred and whether the patches could be characterised by the species they supported. To do this, we used correspondence analysis [48] using a matrix of reporting rates for each species at each site. We defined reporting rate as the number of survey points out of six (three survey points×two repeats) that each species was observed. The correspondence analysis thus characterised the bird community structure at each woodland patch by the identity of the bird species as well as the number of times each species was observed. Second, we related the bird community structure to the woodland vegetation and surrounding woody vegetation variables and the size of the woodland patch. To do this, we employed canonical correspondence analysis [49]. We started with the full ordination model that included all variables; these were scaled and log-transformed prior to inclusion in the model. We then used permutation tests, with a maximum of 1000 permutations, to test the significance of the marginal effects of the individual variables. We successively removed least-significant variables until all variables remaining in the model were significant (P≤0.05). We then tested the significance of the first two axes of the ordination (P≤0.05). We plotted the relationship between individual bird species and the vegetation variables in the final model for 2002 and 2008 combined, 2002 only and 2008 only, and identified species of conservation concern (sensu [11]; Table 1).

#### Bird species traits (Question 3)

We assigned a foraging method and nest site trait to each species (Table 2, Table S1 [50]). We used RLQ analysis [51] to explore the underlying relationships between these ecological traits and the woodland vegetation and surrounding woody vegetation variables. For 2002 and 2008 combined, 2002 only, and 2008 only, we used the woodland vegetation and surrounding woody vegetation variables identified in the final canonical correspondence analysis models (see above). We plotted the ordination and grouped species traits by their relationship with the vegetation variables.

**Table 2:** Species of conservation concern (listed as a declining woodland species by Watson [30] and/or listed in national and state-level threatened species legislation).

Code	Name	Scientific Name	Foraging Method	Nest Site
BCH	Black-chinned Honeyeater	*Melithreptus gularis*	Foliage Search	Foliage
BTr	Brown Treecreeper	*Climacteris picumnus*	Wood Search	Hollow
CST	Crested Shrike-tit	*Falcunculus frontatus*	Wood Search	Fork or Branch
DF	Diamond Firetail	*Stagonopleura guttata*	Granivore	Foliage
DW	Dusky Woodswallow	*Artamus cyanopterus*	Hawk/Sally	Fork or Branch
EYR	Eastern Yellow Robin	*Eopsaltria australis*	Pounce	Fork or Branch
GCB	Grey-crowned Babbler	*Pomatostomus temporalis*	Ground Carnivore/Forage	Foliage
HR	Hooded Robin	*Melanodryas cucullata*	Pounce	Fork or Branch
JW	Jacky Winter	*Microeca fascinans*	Hawk/Sally	Fork or Branch
RCR	Red-capped Robin	*Petroica goodenovii*	Pounce	Fork or Branch
ReF	Restless Flycatcher	*Myiagra inquieta*	Hawk/Sally	Fork or Branch
RuW	Rufous Whistler	*Pachycephala rufiventris*	Wood Search	Foliage
SoW	Southern Whiteface	*Aphelocephala leucopsis*	Ground Carnivore/Forage	Hollow
SuP	Superb Parrot	*Polytelis swainsonii*	Granivore	Hollow
WBB	White-browed Babbler	*Pomatostomus superciliosus*	Ground Carnivore/Forage	Foliage
WBroW	White-browed Woodswallow	*Artamus superciliosus*	Hawk/Sally	Fork or Branch

## Results

We found that several of the vegetation variables differed significantly between 2002 and 2008. The amount of tree dieback in the woodland patches increased significantly between these two periods, as did the canopy depth (Table 1; Fig. S1). The number of strata and the midstorey cover occurrence decreased significantly. The other woodland vegetation attributes did not significantly differ between the two years. In the surrounding landscape, percent woody vegetation cover increased significantly at all three scales of measurement (Table 1). This increase primarily reflects growth of vegetation within existing revegetation plantings and woodland patches to the 20% minimum canopy cover, 0.2 ha area and 2 m height thresholds to be classified as vegetation cover in 2008 compared with 2002.

We recorded 92 species of birds in 2002 and 2008, excluding waterbirds (Table S1). Our surveys showed a very high level of thoroughness, with the number of species observed ranging between 90.12% and 99.91% of estimated richness (Table S2). We recorded 87 species at ≥2 sites in 2002 and 2008 combined, 70 species at ≥2 sites in 2002, and 80 species at ≥2 sites in 2008. Of the 17 species of conservation concern observed during the study period (Table S1, Fig. S2), 16 were recorded at ≥2 sites in one or both years (Table 2). The Speckled Warbler (*Chthonicola sagittata*) was recorded at only one site in 2002 and 2008 and was excluded from analysis.

### Bird Community Composition (Questions 1 and 2)

We found strong structure in the bird community in 2002 and 2008 combined (first canonical correlation: 0.67), 2002 only (first canonical correlation: 0.69) and 2008 only (first canonical correlation: 0.67). Bird community structure could be explained by a combination of woodland vegetation and landscape context (Table 3). These drivers differed slightly between 2002, 2008 and both years combined, but there was overall consistency in how they shaped the bird community.

**Table 3:** Canonical correspondence analysis results for the final model for 2002 and 2008 combined, 2002 only and 2008 only.

Year	Final model		Df	χ^2^	F	Pr(>F)	Eig.	Prop.
2002 and 2008 combined	Variables	Leaf litter	1	0.03	1.51	0.032		
		Canopy depth	1	0.03	1.50	0.032		
		Hollow-bearing trees	1	0.03	1.50	0.036		
		Mistletoe occurrence	1	0.06	2.53	0.001		
		Patch size	1	0.04	1.96	0.002		
		Woody vegetation cover, 100 ha	1	0.04	1.81	0.010		
		Woody vegetation cover, 1,000 ha	1	0.06	2.76	0.001		
		Woody vegetation cover, 10,000 ha	1	0.12	5.23	0.001		
		Year	1	0.05	2.21	0.003		
	Axes	Axis 1	1	0.20	8.83	0.001	0.20	0.39
		Axis 2	1	0.07	2.99	0.001	0.07	0.13
2002 only	Variables	Dieback score	1	0.06	1.59	0.040		
		Mistletoe occurrence	1	0.09	2.30	0.001		
		Woody vegetation cover, 1,000 ha	1	0.09	2.42	0.002		
		Woody vegetation cover, 10,000 ha	1	0.14	3.64	0.001		
	Axes	Axis 1	1	0.22	5.83	0.001	0.22	0.58
		Axis 2	1	0.07	1.86	0.016	0.07	0.18
2008 only	Variables	Leaf litter	1	0.06	1.69	0.010		
		Mistletoe occurrence	1	0.08	2.13	0.002		
		Patch size	1	0.07	1.89	0.004		
		Woody vegetation cover, 100 ha	1	0.06	1.65	0.026		
		Woody vegetation cover, 1,000 ha	1	0.08	2.09	0.001		
		Woody vegetation cover, 10,000 ha	1	0.13	3.40	0.001		
	Axes	Axis 1	1	0.20	5.32	0.001	0.20	0.41
		Axis 2	1	0.09	2.34	0.001	0.09	0.18

Variables: All variables in each model had significant marginal effects. Axes: The first two axes in each model were significant, and the eigenvalues (Eig.) and proportion of variance explained (Prop.) are given.

**Figure 2:**
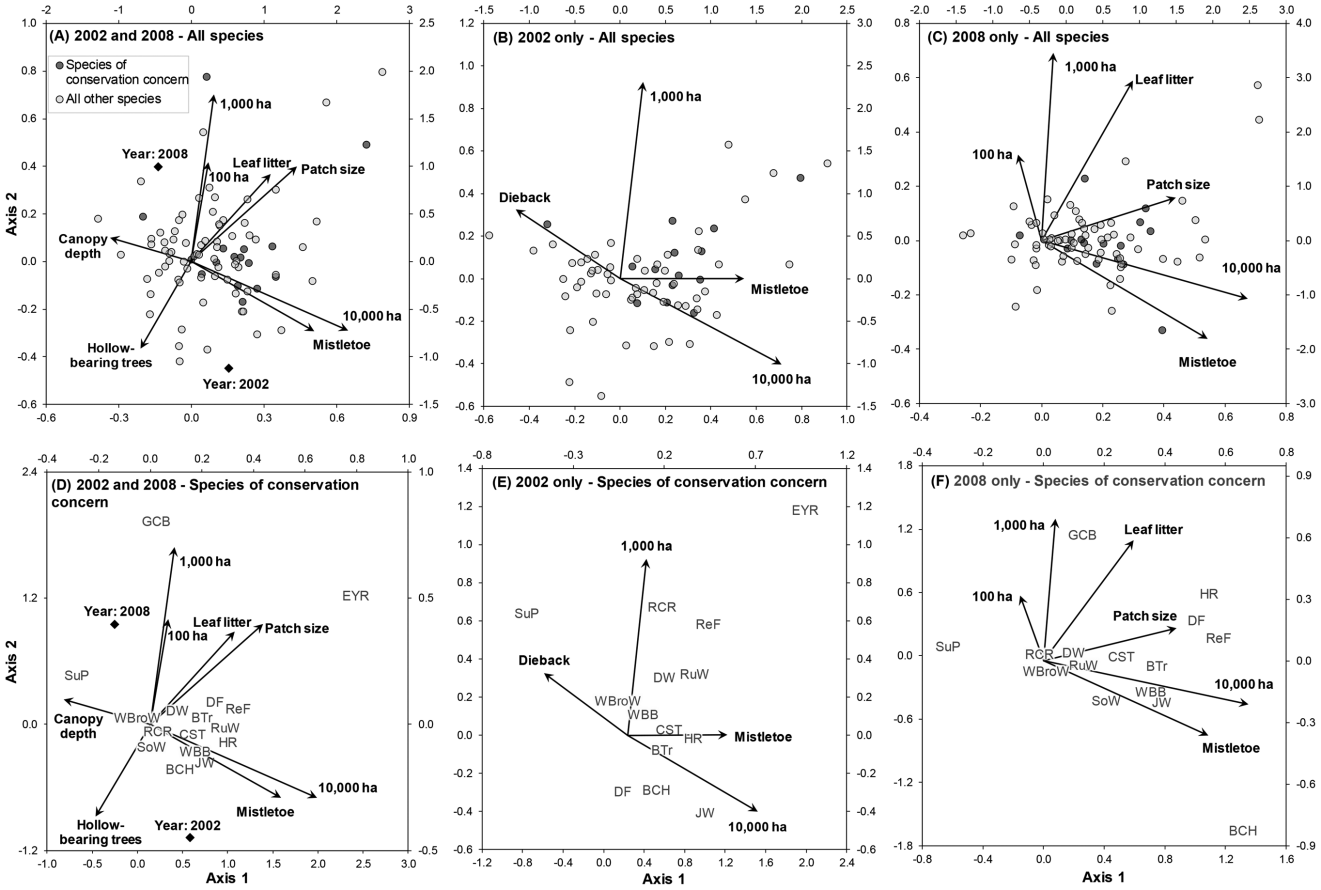
Ordinations of the final canonical correspondence analysis models showing relationship between bird species and woodland patch vegetation and surrounding woody vegetation variables. All species plotted, with species of conservation concern identified: (A) both years combined, (B) 2002, and (C) 2008. Only species of conservation concern plotted: (D) both years combined, (E) 2002, and (F) 2008. See Table S1 for full list of species included in the analyses. See Table 2 for species codes.

In 2002 and 2008 combined, we found that woodland patch leaf litter cover, canopy depth, hollow bearing tree density, mistletoe occurrence, patch size, and surrounding woody vegetation cover at all three scales (100 ha, 1,000 ha and 10,000 ha) significantly affected community composition (Fig. 2A, Table 3). Community composition also was significantly different between years. Axis 1 explained 39% of variation and arranged sites from those with trees with large canopies and high numbers of hollow bearing trees to those sites within larger patches with high leaf litter cover, mistletoe occurrence and high woody vegetation cover at the 10,000 ha scale. All species of conservation concern were positively associated with this axis, with the exception of the Superb Parrot (see Table 2 for scientific names), which was positively associated with canopy depth (Fig. 2D). Axis 2 explained 15% of variance and arranged sites from those with high woody vegetation cover at the 10,000 ha scale to those with high woody vegetation cover at the 100 ha and 1,000 ha scales. Species of conservation concern were associated with both scales of vegetation cover. In particular, the Grey-crowned Babbler was associated with woody vegetation cover at the 1,000 ha scale. Axis 2 also differentiated the bird community between 2002 and 2008; however no species, including those of conservation concern, were strongly associated with either year.

In 2002, we found that woodland patch dieback, mistletoe occurrence, and surrounding woody vegetation cover at the 1,000 ha and 10,000 ha scales significantly affected community composition (Fig. 2B, Table 3). Axis 1 explained 58% of variation and arranged sites from those with high dieback scores to those with mistletoe and high woody vegetation cover at the 10,000 ha scale. All species of conservation concern were positively associated with this axis, with the exception of the Superb Parrot (Fig. 2E). In particular, the Hooded Robin appeared to be positively associated with mistletoe and the Jacky Winter with woody vegetation cover at the 10,000 ha scale. Axis 2 explained 18% of variance and arranged sites from those with high woody vegetation cover at the 10,000 ha scale to those with high woody vegetation cover at the 1,000 ha scale. Species of conservation concern were associated with both scales of cover.

In 2008, we found that woodland patch leaf litter cover, mistletoe occurrence, patch size, and surrounding woody vegetation cover at all three scales (100 ha, 1,000 ha and 10,000 ha) significantly affected community composition (Fig. 2C, Table 3). Axis 1 explained 41% of variance and arranged sites along a gradient of increasing patch size, mistletoe occurrence, leaf litter cover and woody vegetation cover at the 10,000 ha scale. Similar to 2002, all species of conservation concern, with the exception of the Superb Parrot, were positively associated with this axis (Fig. 2F). Axis 2 explained 18% of variance and arranged sites along a gradient of increasing leaf litter cover and woody vegetation cover at the 100 ha and 1,000 ha scales. Again, species of conservation concern were associated with all scales of surrounding woody vegetation cover.

### Bird Species Traits (Question 3)

**Figure 3:**
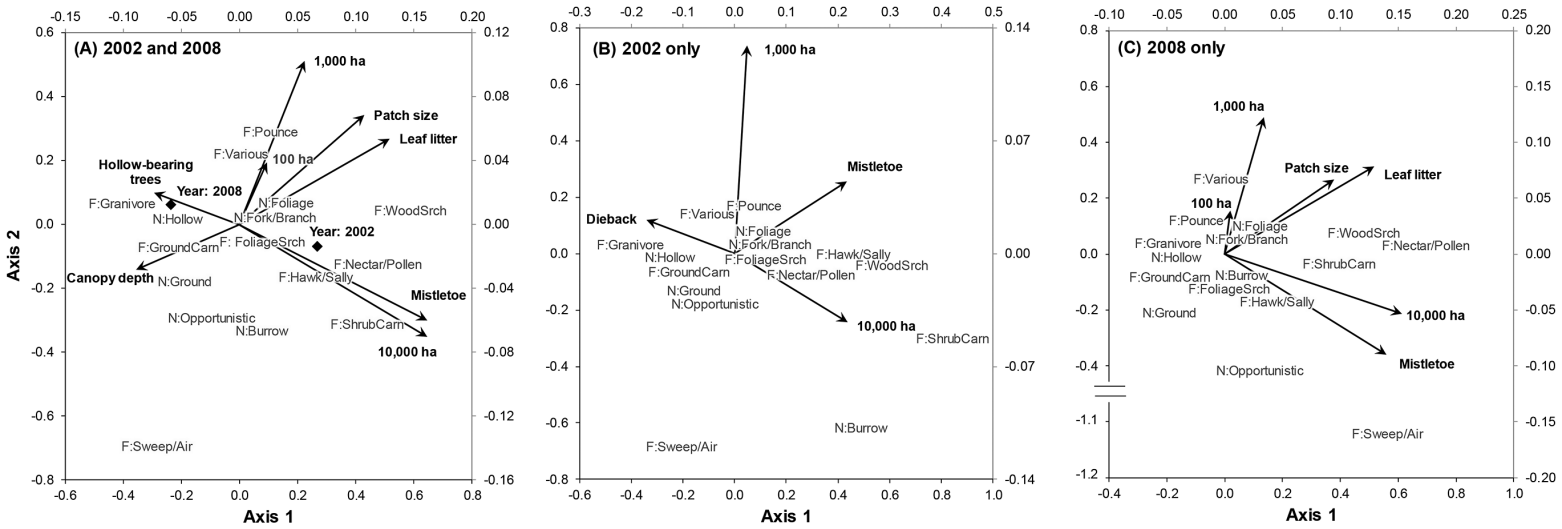
Ordinations of the RLQ analyses for: (A) both years combined, (B) 2002, and (C), 2008, showing foraging method (F) and nest site (N) traits. See Table S1 for the full list of species included in the analysis and their assigned traits.

We found that the relationships between the bird community and woodland patch and surrounding vegetation cover were underpinned by significant relationships between the ecological traits of the bird community and the woodland vegetation and surrounding woody vegetation variables (Table 4, see below). Moreover, similar to our findings for bird community composition, there was overall consistency in the relationships between the two survey years (Fig. 3).

**Table 4:** Results of RLQ analyses of the vegetation cover within and surrounding each woodland patch (R), the species present (L), and their life-history traits (Q) for 2002 and 2008 combined, 2002 only and 2008 only.

		2002 and 2008 combined		2002		2008	
		Axis 1 (%)	Axis 2 (%)	Axis 1 (%)	Axis 2 (%)	Axis 1 (%)	Axis 2 (%)
Separate ordinations	R(PCA)	1.80(19.98)	1.55 (17.26)	1.46 (36.39)	1.04 (25.97)	1.75 (29.23)	1.37 (22.91)
	L(CA)	0.45 (9.36)	0.26 (5.45)	0.48 (11.64)	0.34 (8.34)	0.45 (11.12)	0.23 (5.64)
	Q(PCA)	1.80 (12.87)	1.70 (12.15)	1.89 (10.48)	1.80 (9.98)	1.79 (12.80)	1.71 (12.18)
RLQ analysis	RLQ axis eigenvalues	0.10 (57.28)	0.03 (16.75)	0.16 (81.59)	0.02 (10.19)	0.07 (53.56)	0.04 (29.19)
	Covariance	0.32	0.17	0.40	0.14	0.26	0.19
	Correlation: L	0.23 (34.49)	0.14 (26.87)	0.32 (46.77)	0.13 (21.36)	0.21 (31.87)	0.15 (31.44)
	Projected variance: R	1.43 (79.38)	2.92 (87.26)	1.16 (79.96)	2.23 (89.30)	1.25 (71.56)	2.80 (89.34)
	Projected variance: Q	1.32 (73.00)	2.35 (67.18)	1.33 (70.72)	2.55 (69.30)	1.22 (68.33)	2.33 (66.53)

Separate ordinations: eigenvalues (and percent variance explained) for the first two axes from the ordinations of the R (Hill-Smith principal components analysis), L (correspondence analysis) and Q (Hill-Smith principal components analysis) tables. RLQ analysis: eigenvalues (and percent variance explained), covariance and correlation (and percent variance) with the correspondence analysis of the L matrix, and projected variance (and percent variance) with the R and Q matrices.

We found that species that forage using foliage search, nectar/pollen collector, hawk/sally and wood search/bark probe methods were associated with larger woodland patches with mistletoe and high levels of leaf litter and woody vegetation cover at the 10,000 ha scale (Fig. 3). Of the 16 species of conservation concern analysed, eight shared these foraging traits (Table 2). Species that pounce or employ a variety of methods were associated with woodland patches with levels of high woody vegetation cover at the 100 ha and 1,000 ha scales; three species of conservation concern were pounce foragers. Also associated with these scales were species that nest in foliage or in tree forks and branches. All but three species of conservation concern shared these traits. In contrast, we found that species that forage using sweep/air pursuit methods or that select nest sites opportunistically or on the ground were associated with woodland patches with low levels of woody vegetation cover in the surrounding landscape at all scales. Lastly, we found that ground carnivores/foragers, granivores and hollow-nesters were associated with woodland patches with trees with large canopies, high dieback scores and numbers of hollow-bearing trees and low leaf litter cover and mistletoe occurrence.

## Discussion

We investigated multi-scale associations between vegetation cover and woodland bird communities across a large agricultural region. We found that both woodland patch vegetation and surrounding woody vegetation cover were important for structuring the bird community and that there was consistency between these scales over time (*Question 1*). In particular, the occurrence of mistletoe within the woodland patches and high levels of woody vegetation cover at the 1,000 ha and 10,000 ha scales were important, especially for species of conservation concern. We found that these species displayed similar responses to the patch and landscape vegetation cover (*Question 2*). We also found that these relationships were related to the foraging and nesting traits of the bird community (*Question 3*). As we discuss below, these findings confirm those from previous studies of species-specific, species-diversity and community composition responses to vegetation cover and structure. However, the spatial scale of our study affords greater power than most previous studies and enables us to make strong inferences about patch and landscape-scale determinants of woodland suitability for a wide range of species.

### Bird Community Composition Associations with Patch and Landscape-scale Vegetation

The final canonical correspondence models of the bird community all included a combination of patch-scale and landscape-scale vegetation variables. These results are consistent with recent studies investigating patch and matrix effects on bird communities [10], [18]–[20]. These previous studies have mostly focused on contrasting patch types (e.g. riparian vs. non-riparian vs. pasture [10]) or contrasting matrix types (e.g. vegetated vs. open agricultural [19]). Our study, in comparison, showed that fine-scale differences in both patch vegetation structure and landscape vegetation cover also have important effects on bird communities. These findings support detailed studies of individual species that show that small differences in habitat quality can influence patch occupancy and abundance (e.g. [11], [52], [53]). Species-specific perceptions of habitat quality arise from individual foraging and nesting requirements, as well as dispersal limitations [54]. The brown treecreeper, for example, forages and nests among coarse woody debris but also requires high habitat connectivity in the landscape due to the limited dispersal ability of the females [55]–[57]. The individual responses of each species within the bird community combine to shape community-level responses to patch and landscape-scale vegetation [17].

Most bird species of conservation concern had similar associations with the patch and landscape-scale vegetation. We found that the presence of mistletoe was particularly important, lending further support to its role as a keystone resource [58]. Mistletoe provides high quality foraging and nesting resources, and is important for many guilds of birds and mammals [58]. In addition to the direct provision of fruit and nectar resources for birds, mistletoe leaf litter supports abundant arthropod communities which can be an important food source [59]. For instance, the species of conservation concern that we investigated use a number of foraging strategies but all include invertebrates, exclusively or occasionally, in their diet. Our findings support those of Watson and Herring [59], who found that the experimental removal of mistletoe led to significant decreases in woodland dependent and resident bird species, with changes seen across the whole bird community.

The bird community was significantly associated with woodland patch size in 2008 and when considering both years combined. Patch size is considered a key correlate with species occupancy and diversity [60], [61], with larger patches having higher occupancy and supporting more species. For example, Díaz et al. [62] found that patch size explained 67–75% of variation in bird species richness in pine plantations. Patch size, however, may be confounded with habitat loss and fragmentation [63], and the effect of patch size may be related to vegetation type [64] and matrix conditions [65]. Furthermore, a recent meta-analysis [66] found that patch size, whilst important, was a poor predictor of species occupancy. In our study, the bird community associated with patch size was similarly associated with patch and landscape-scale vegetation cover. Thus, we were unable to identify the independent importance of patch-size for structuring the bird community.

The period of our study coincided with the “Millennium Drought”, the most severe drought Australia has experienced since 1900 [40], and it is likely that this drought affected the bird community. Direct effects of drought on fauna include altered trophic relationships, range shifts and novel species associations (reviewed by [67]–[69]). Indirect effects include tree death and dieback [41] and disruptions to pollination [70]. Underlying some of these impacts is the reduction or change in the availability of habitat resources across space and in time. Critically, in landscapes where habitat resources have already been depleted due to other forms of landscape change (such as caused by agriculture) extreme climatic events can exacerbate pressures already experienced by species dependent on remnant vegetation, rendering them more sensitive to future change [32], [67]. Further, inter-regional differences in fragmentation and climate may lead to some species increasing or decreasing in parts of their range [26], and subsequent shifts in community composition. Given the expected changes in climate and rainfall patterns in many parts of the world [71], [72], the relative importance of remnant vegetation for supporting fauna may increase. From a conservation management perspective, it is crucial that we better understand what influences species occurrence and community composition within fragmented woodland vegetation during extreme climatic events. Our finding of consistent relationships between the bird community and patch and landscape-scale vegetation during the drought period suggests that management actions focused at these scales are likely to continue to be important under future climate scenarios.

### Bird Species Trait Associations with Patch and Landscape-scale Vegetation

Our work demonstrates how investigating underlying ecological traits gives greater insights into community patterns [37], [38]. For instance, we found a clear separation between species that forage or nest in vegetation (e.g. shrub carnivores and branch nesters) and those that are able to use more open areas (e.g. sweep/air pursuit feeders and ground nesters). This indicates that the loss or gain of vegetation structure in woodland patches will have an impact upon specific components of the bird community. We found that species of conservation concern shared similar foraging and nesting traits, but represented several foraging and nesting guilds. This agrees with similar results from previous studies [26], [32] and supports the maintenance of heterogeneous habitat containing diverse resource niches [31]. Several previous studies have suggested that ground-foraging insectivores are in decline [25], [30]. However, we did not find an association between ground-foraging and species of conservation concern. We note, however, that this guild included several ‘open country’ species, for example the Magpie Lark (*Grallina cyanoleuca)* and Australasian Pipit (*Anthus novaeseelandiae*), and their association with less structurally-dense vegetation may have influenced our findings.

### Management Implications

Similarities among bird species of conservation concern in their relationships to patch and landscape-scale vegetation cover suggest that management strategies aimed at individual species are likely to have wider benefits for other species. In the remainder of this paper, we discuss management strategies to increase woodland patch vegetation quality and the cover of surrounding woody vegetation in fragmented agricultural landscapes to achieve the improved conservation of woodland bird communities. We advise that these management strategies be implemented under an adaptive monitoring framework [73] to assess their outcomes.

#### 1. Woodland condition

The presence of mistletoe in the woodland patches (irrespective of its abundance) was important for structuring the bird community, and associated with species of conservation concern. As such, we recommend that greater consideration be given to the maintenance and perpetuation of mistletoe in agricultural landscapes. Management may have to be undertaken indirectly, however, because mistletoe cannot be transplanted and inoculation is difficult to achieve [58]. Instead, management approaches aimed at increasing woodland condition may be more effective [74]: higher quality remnants may attract the bird species capable of dispersing mistletoe seeds and improved tree health will enable the deposited seeds to grow and mature.

#### 2. Structural diversity

In contrast to other species of conservation concern, the Superb Parrot was associated with sites with large canopies and hollow-bearing trees, reflecting its distinct habitat preferences. Large, old living and dead trees provide hollows crucial for the Superb Parrot and other hollow-nesting species [75], and woodland patches with dense stands of smaller or younger trees do not provide equivalent resources. The Superb Parrot thus serves as a reminder that it is important to have structural diversity across woodland patches, i.e. “don’t have the same thing everywhere” [76].

#### 3. Regrowth vegetation

We found that the cover of woody vegetation in the surrounding landscape was associated with bird community composition. Woodland patches supported more species of conservation concern when in landscapes with high woody vegetation cover at the 1,000 ha and 10,000 ha scales. Landscape-scale vegetation cover may buffer changes in patch-scale vegetation cover, and measures to increase native vegetation cover in agricultural landscapes are vital to improved conservation outcomes [77]. A potential focus for management interventions includes increasing/preserving stands of native regrowth, which provides habitat for a range of species [11], [77], including many woodland birds. It is therefore important that regrowth receives sufficient formal protection. For example, in our study region, native vegetation that has regenerated since 1990 is classified as regrowth and is regulated by government legislation on tree clearing (Native Vegetation Act 2003). Proposed changes to this legislation, however, will allow ‘thinning’ of dense vegetation such as regrowth. This raises concerns for the structural integrity of regrowth and its associated benefits for woodland birds.

#### 4. Revegetation plantings

Another widely applied management intervention to increase landscape vegetation cover in agricultural landscapes worldwide [78], and in southeastern Australia in particular [20], is to actively revegetate areas. This approach provides important habitat for woodland birds, including many of conservation concern [56], [79], [80], and may be an important adaptation to climate change [81]. However, extreme climatic events, such as drought, can be detrimental to the success of restoration efforts [82], and it is critical that revegetation programs consider these potential impacts. One measure to improve the success of revegetation plantings is to choose plant species capable of establishing and surviving drought [81].

## Conclusions

Improving biodiversity conservation in fragmented agricultural landscapes has become an important global issue [83]. This is evident through the large investments in farmland biodiversity that are becoming increasingly common (e.g. agri-environmental schemes [84], [85]). Missing from much of the ecological research underpinning these schemes, however, are investigations at the level of the community assemblage [17]. Addressing this knowledge gap improves our ability to generalise across agricultural landscapes, and leads to integrated multi-species conservation policies and management. Our investigation of multi-scale associations between vegetation cover and woodland bird communities shows that both patch-scale vegetation structure and landscape-scale vegetation cover are important determinants of community composition. This finding supports those from previous species-specific and species diversity research, and from different regions worldwide [13]–[15], [86]. Further, species of conservation concern showed similar responses. This suggests that the species under most threat in agricultural landscapes will be positively affected by undertaking management actions to improve woodland condition and landscape vegetation cover.

## Supporting Information

Figure S1 **Mean values (± standard error) for remnant vegetation and surrounding woody vegetation variables in 2002 and 2008.** Paired t-tests were used to test for significant differences (P≤0.003) between the two study years.
(TIF)

Figure S2 **Some of the bird species of conservation concern analysed in this study.** Clockwise from top left: Pair of hooded robins, grey-crowned babbler, brown treecreeper, eastern yellow robin and immature red-capped robin. Scientific names are given in Table S1. Images by D. Stojanovic.
(TIF)

Table S1 **Full list of 92 bird species recorded in study (excluding waterbirds), including species of conservation concern (declining woodland species and/or listed in national and state-level threatened species legislation).** In 2002 and 2008 combined, 92 species were recorded, of which five species were recorded at 1 site (∼) and 87 were recorded at ≥2 sites (*) In 2002, 83 species were recorded, of which 70 were recorded at ≥2 sites. In 2008, 86 species were recorded, of which 80 were recorded at ≥2 sites. Key to foraging method: F = foliage search, G = granivore, GCF = ground carnivore or forage, HS = hawk/sally, NP = nectar/pollen collection, P = pounce, SAP = sweep/air pursuit, SC = shrub carnivore, VM = various methods, and WBS = wood/bark search. Key to nest site: B = burrow, F = foliage, FB = fork or branch, G = ground, H = hollow, and O = opportunistic.
(DOCX)

Table S2 **Summary of the number of observed bird species, and estimated species richness in 2002 and 2008.**
(DOCX)
