# Supplementary figures and images for: Multi-Scale Associations between Vegetation Cover and Woodland Bird Communities across a Large Agricultural Region
Source: PLoS One. 2014 May 15;9(5):e97029. doi: 10.1371/journal.pone.0097029 (PMC4022507; doi:10.1371/journal.pone.0097029)

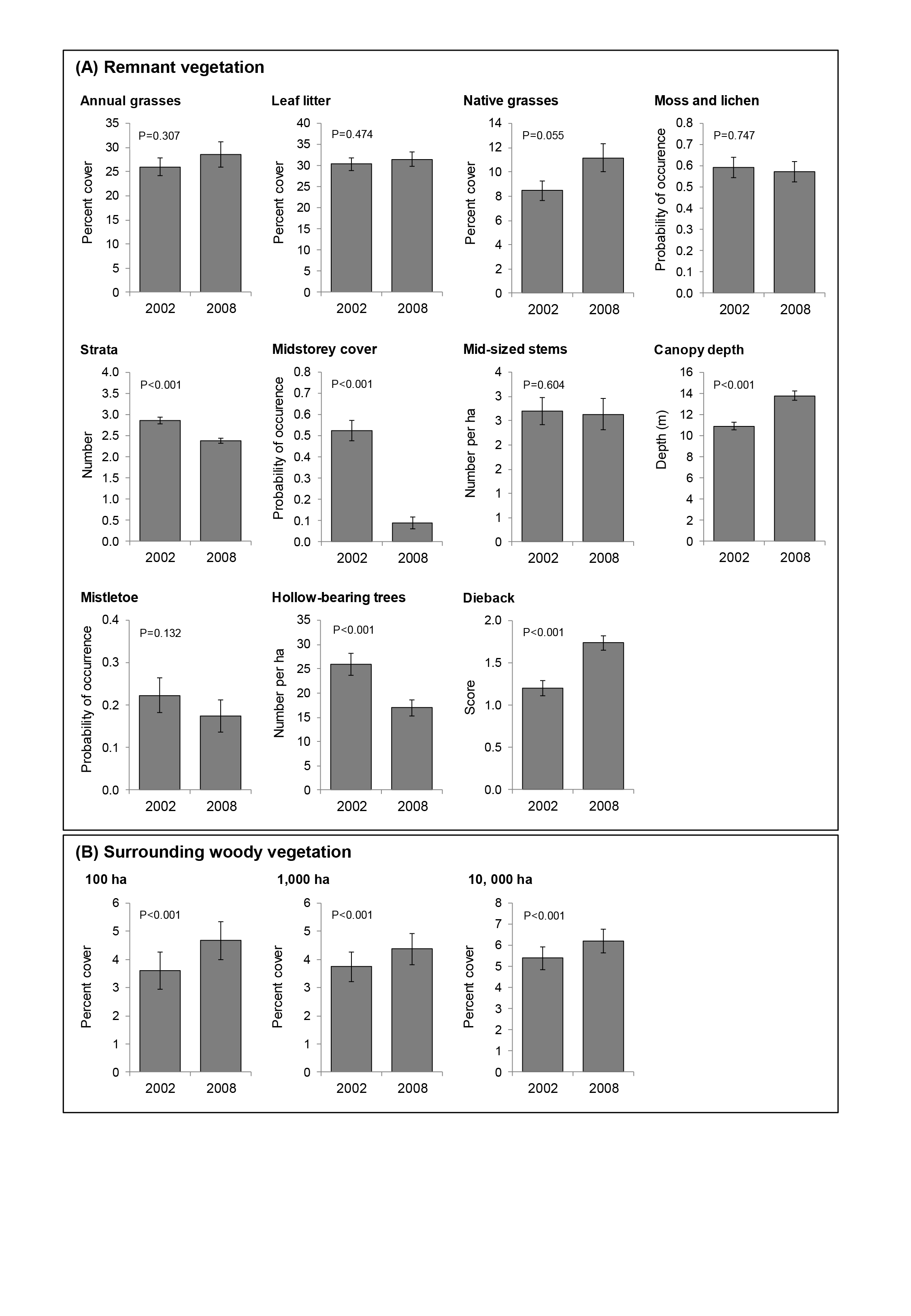

Supplement: Figure S1 — Mean values (± standard error) for remnant vegetation and surrounding woody vegetation variables in 2002 and 2008. Paired t-tests were used to test for significant differences (P≤0.003) between the two study years. (TIF) [file pone.0097029.s001.tif]

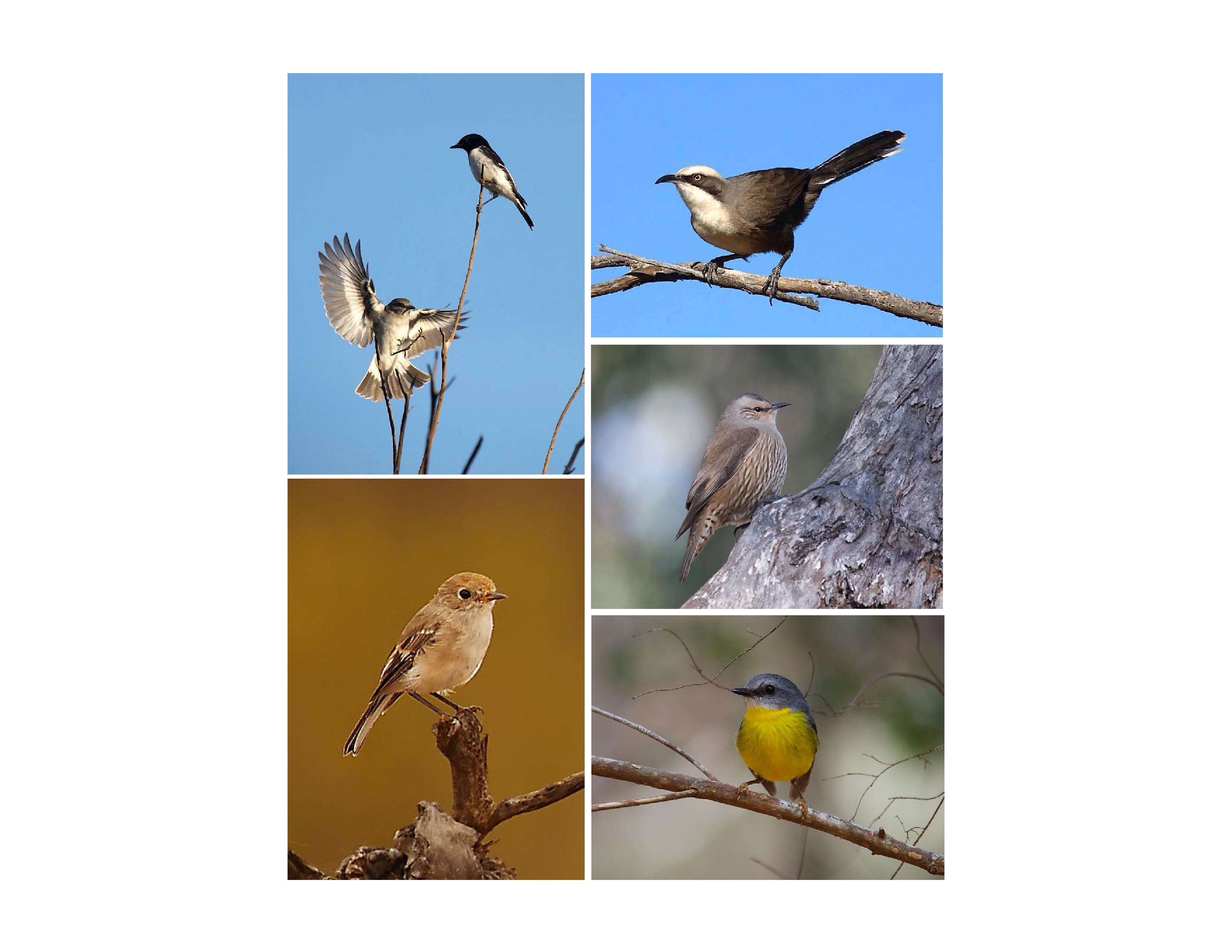

Supplement: Figure S2 — Some of the bird species of conservation concern analysed in this study. Clockwise from top left: Pair of hooded robins, grey-crowned babbler, brown treecreeper, eastern yellow robin and immature red-capped robin. Scientific names are given in Table S1. Images by D. Stojanovic. (TIF) [file pone.0097029.s002.tif]
