# Supplementary material for: Multi-Scale Associations between Vegetation Cover and Woodland Bird Communities across a Large Agricultural Region
Source: PLoS One. 2014 May 15;9(5):e97029. doi: 10.1371/journal.pone.0097029 (PMC4022507; doi:10.1371/journal.pone.0097029)
Supplement: Table S1 — Full list of 92 bird species recorded in study (excluding waterbirds), including species of conservation concern (declining woodland species and/or listed in national and state-level threatened species legislation). In 2002 and 2008 combined, 92 species were recorded, of which five species were recorded at 1 site (∼) and 87 were recorded at ≥2 sites (*) In 2002, 83 species were recorded, of which 70 were recorded at ≥2 sites. In 2008, 86 species were recorded, of which 80 were recorded at ≥2 sites. Key to foraging method: F = foliage search, G = granivore, GCF = ground carnivore or forage, HS = hawk/sally, NP = nectar/pollen collection, P = pounce, SAP = sweep/air pursuit, SC = shrub carnivore, VM = various methods, and WBS = wood/bark search. Key to nest site: B = burrow, F = foliage, FB = fork or branch, G = ground, H = hollow, and O = opportunistic. (DOCX) [file pone.0097029.s003.docx]

**Table S1. Full list of 92 bird species recorded in study (excluding waterbirds), including species of conservation concern (declining woodland species and/or listed in national and state-level threatened species legislation).** In 2002 and 2008 combined, 92 species were recorded, of which five species were recorded at 1 site (~) and 87 were recorded at ≥2 sites (*) In 2002, 83 species were recorded, of which 70 were recorded at ≥2 sites. In 2008, 86 species were recorded, of which 80 were recorded at ≥2 sites. Key to foraging method: F = foliage search, G = granivore, GCF = ground carnivore or forage, HS = hawk/sally, NP = nectar/pollen collection, P = pounce, SAP = sweep/air pursuit, SC = shrub carnivore, VM = various methods, and WBS = wood/bark search. Key to nest site: B = burrow, F = foliage, FB = fork or branch, G = ground, H = hollow, and O = opportunistic.

| Name | Scientific Name | Foraging Method | Nest Site | Cons. Concern | 2002 and 2008 | 2002 only | 2008 only |
| --- | --- | --- | --- | --- | --- | --- | --- |
| Apostlebird | *Struthidea cinerea* | GCF | FB |  | * | ^~^ | ^~^ |
| Australasian Pipit | *Anthus novaeseelandiae* | GCF | G |  | * | * | * |
| Australian Magpie | *Cracticus tibicen* | GCF | FB |  | * | * | * |
| Australian Raven | *Corvus coronoides* | VM | FB |  | * | * | * |
| Black-chinned Honeyeater | *Melithreptus gularis* | F | F | * | * | * | * |
| Black-faced Cuckoo-shrike | *Coracina novaehollandiae* | WBS | FB |  | * | * | * |
| Blue-faced Honeyeater | *Entomyzon cyanotis* | SC | F |  | * | * | * |
| Brown Falcon | *Falco berigora* | P | O |  | ^~^ | ^~^ |  |
| Brown Goshawk | *Accipiter fasciatus* | P | FB |  | * | ^~^ | ^~^ |
| Brown Songlark | *Cincloramphus cruralis* | GCF | G |  | * | * | * |
| Brown Treecreeper | *Climacteris picumnus* | WBS | H | * | * | * | * |
| Brown-headed Honeyeater | *Melithreptus brevirostris* | F | F |  | * | ^~^ | * |
| Budgerigar | *Melopsittacus undulatus* | G | H |  | * |  | * |
| Buff-rumped Thornbill | *Acanthiza reguloides* | SC | F |  | * | * | * |
| Cockatiel | *Nymphicus hollandicus* | G | H |  | * | * | * |
| Common Blackbird | *Turdus merula* | GCF | F |  | * | * | * |
| Common Bronzewing | *Phaps chalcoptera* | G | FB |  | * | * | * |
| Common Starling | *Sturnus vulgaris* | GCF | O |  | * | * | * |
| Crested Pigeon | *Ocyphaps lophotes* | G | FB |  | * | * | * |
| Crested Shrike-tit | *Falcunculus frontatus* | WBS | FB | * | * | * | * |
| Crimson Rosella | *Platycercus elegans* | VM | H |  | * | * | * |
| Diamond Firetail | *Stagonopleura guttata* | G | F | * | * | * | * |
| Dollarbird | *Eurystomus orientalis* | HS | H |  | * | * | * |
| Dusky Woodswallow | *Artamus cyanopterus* | HS | FB | * | * | * | * |
| Eastern Rosella | *Platycercus eximius* | G | H |  | * | * | * |
| Eastern Yellow Robin | *Eopsaltria australis* | P | FB | * | * | * | ^~^ |
| Fairy Martin | *Petrochelidon ariel* | SAP | O |  | * | * |  |
| Fuscous Honeyeater | *Lichenostomus fuscus* | F | F |  | * | * | * |
| Galah | *Eolophus roseicapillus* | G | H |  | * | * | * |
| Golden Whistler | *Pachycephala pectoralis* | WBS | F |  | ^~^ | ^~^ |  |
| Grey Butcherbird | *Cracticus torquatus* | P | FB |  | * | * | * |
| Grey Fantail | *Rhipidura albiscapa* | HS | FB |  | * | * | * |
| Grey Shrike-thrush | *Colluricincla harmonica* | VM | O |  | * | * | * |
| Grey-crowned Babbler | *Pomatostomus temporalis* | GCF | F | * | * |  | * |
| Hooded Robin | *Melanodryas cucullata* | P | FB | * | * | * | * |
| Horsfield's Bronze-Cuckoo | *Chalcites baslis* | VM | F |  | * | * | * |
| House Sparrow | *Passer domesticus* | VM | O |  | * | * | * |
| Jacky Winter | *Microeca fascinans* | HS | FB | * | * | * | * |
| Laughing Kookaburra | *Dacelo novaeguineae* | P | H |  | * | * | * |
| Little Corella | *Cacatua sanguinea* | G | H |  | * | ^~^ | * |
| Little Friarbird | *Philemon citreogularis* | NP | F |  | * | * | * |
| Little Lorikeet | *Glossopsitta pusilla* | NP | H |  | * | * | * |
| Little Raven | *Corvus mellori* | GCF | FB |  | * |  | * |
| Magpie-lark | *Grallina cyanoleuca* | GCF | FB |  | * | * | * |
| Masked Lapwing | *Vanellus miles* | GCF | G |  | * | ^~^ | ^~^ |
| Masked Woodswallow | *Artamus personatus* | HS | FB |  | * | * | * |
| Mistletoebird | *Dicaeum hirundinaceum* | F | F |  | * | * | * |
| Nankeen Kestrel | *Falco cenchroides* | P | H |  | * |  | * |
| Noisy Friarbird | *Philemon corniculatus* | NP | F |  | * | * | * |
| Noisy Miner | *Manorina melanocephala* | VM | F |  | * | * | * |
| Olive-backed Oriole | *Oriolus sagittatus* | WBS | F |  | * | * | * |
| Pallid Cuckoo | *Cacomantis pallidus* | VM | FB |  | * |  | * |
| Peaceful Dove | *Geopelia striata* | G | FB |  | * | * | * |
| Peregrine Falcon | *Falco peregrinus* | SAP | O |  | ^~^ | ^~^ |  |
| Pied Butcherbird | *Cracticus nigrogularis* | P | FB |  | * | * | * |
| Pied Currawong | *Strepera graculina* | VM | FB |  | * | * | * |
| Rainbow Bee-eater | *Merops ornatus* | HS | B |  | * | * | * |
| Red Wattlebird | *Anthochaera carunculata* | NP | F |  | * | * | * |
| Red-browed Finch | *Neochmia temporalis* | G | FB |  | ^~^ | ^~^ |  |
| Red-capped Robin | *Petroica goodenovii* | P | FB | * | * | * | * |
| Red-rumped Parrot | *Psephotus haematonotus* | G | H |  | * | * | * |
| Restless Flycatcher | *Myiagra inquieta* | HS | FB | * | * | * | * |
| Rufous Songlark | *Cincloramphus mathewsi* | GCF | G |  | * | * | * |
| Rufous Whistler | *Pachycephala rufiventris* | WBS | F | * | * | * | * |
| Sacred Kingfisher | *Todiramphus sanctus* | P | B |  | * | * | * |
| Silvereye | *Zosterops lateralis* | VM | F |  | * | ^~^ | * |
| Southern Whiteface | *Aphelocephala leucopsis* | GCF | H | * | * |  | * |
| Speckled Warbler | *Chthonicola sagittata* | GCF | H | * | ^~^ | ^~^ |  |
| Spotted Pardalote | *Pardalotus punctatus* | F | B |  | * | * | * |
| Striated Pardalote | *Pardalotus striatus* | F | H |  | * | * | * |
| Striated Thornbill | *Acanthiza lineata* | F | F |  | * |  | * |
| Stubble Quail | *Coturnix pectoralis* | G | G |  | * |  | * |
| Sulphur-crested Cockatoo | *Cacatua galerita* | G | H |  | * | * | * |
| Superb Fairy-wren | *Malurus cyaneus* | SC | F |  | * | * | * |
| Superb Parrot | *Polytelis swainsonii* | G | H | * | * | * | * |
| Tree Martin | *Petrochelidon nigricans* | SAP | H |  | * | * | * |
| Wedge-tailed Eagle | *Aquila audax* | P | FB |  | * | ^~^ | ^~^ |
| Weebill | *Smicrornis brevirostris* | F | F |  | * | * | * |
| Welcome Swallow | *Hirundo neoxena* | SAP | O |  | * | * | * |
| Western Gerygone | *Gerygone fusca* | F | F |  | * | * | * |
| White-browed Babbler | *Pomatostomus superciliosus* | GCF | F | * | * | * | * |
| White-browed Woodswallow | *Artamus superciliosus* | HS | FB | * | * | * | * |
| White-naped Honeyeater | *Melithreptus lunatus* | F | F |  | * | * |  |
| White-plumed Honeyeater | *Lichenostomus penicillatus* | F | F |  | * | * | * |
| White-throated Gerygone | *Gerygone albogularis* | F | F |  | * |  | * |
| White-throated Treecreeper | *Cormobates leucophaea* | WBS | H |  | * | * | * |
| White-winged Chough | *Corcorax melanorhamphos* | GCF | FB |  | * | * | * |
| White-winged Triller | *Lalage sueurii* | WBS | FB |  | * | * | * |
| Willie Wagtail | *Rhipidura leucophrys* | HS | FB |  | * | * | * |
| Yellow Thornbill | *Acanthiza nana* | F | F |  | * |  | * |
| Yellow-faced Honeyeater | *Lichenostomus chrysops* | F | F |  | * | * | * |
| Yellow-rumped Thornbill | *Acanthiza chrysorrhoa* | GCF | F |  | * | * | * |
