# Supplementary material for: Multi-Scale Associations between Vegetation Cover and Woodland Bird Communities across a Large Agricultural Region
Source: PLoS One. 2014 May 15;9(5):e97029. doi: 10.1371/journal.pone.0097029 (PMC4022507; doi:10.1371/journal.pone.0097029)
Supplement: Table S2 — Summary of the number of observed bird species, and estimated species richness in 2002 and 2008. (DOCX) [file pone.0097029.s004.docx]

**Table S2. Summary of the number of observed bird species, and estimated species richness in 2002 and 2008.**

|  | 2002 | 2008 |
| --- | --- | --- |
| Observed number of species | 82 | 85 |
| Estimated richness |  |  |
| Abundance Coverage-based Estimator (ACE) | 87.37 | 85.65 |
| Incidence Coverage-based Estimator (ICE) | 87.35 | 85.64 |
| Chao 1 richness estimator | 88.00 | 85.08 |
| Chao 2 richness estimator | 87.99 | 85.08 |
| First-order Jackknife richness estimator | 90.99 | 87.00 |
| Bootstrap richness estimator | 86.49 | 87.74 |
